# Controlled capnothorax creation for stapled diaphragm resection: a safe maneuver for en-bloc liver and frenic resection without diaphragm opening

**DOI:** 10.1007/s00464-026-12794-6

**Published:** 2026-04-27

**Authors:** Lucas Sabatella, Daniel Aliseda, Pablo Martí-Cruchaga, Gabriel Zozaya, Luis López-Olaondo, Nuria Blanco Asensio, Fernando Rotellar

**Affiliations:** 1 HPB and Liver Transplant Unit, Department of General Surgery., Clinica Universidad de Navarra, University of Navarra. IDISNA, Av. Pío XII, 36, 31008 Pamplona, Spain; 2 Anesthesiology Department, Clinica Universidad de Navarra, University of Navarra, Pamplona, Spain

**Keywords:** Pneumothorax, Capnothorax, Phrenic infiltration, En-bloc diaphragm resection, Liver tumors

## Abstract

**Background:**

Full-thickness diaphragmatic resection during minimally invasive liver surgery remains technically demanding, particularly for tumors located in posterosuperior segments with suspected diaphragmatic invasion. Stapled en-bloc resection avoids pleural opening but is limited by negative intrathoracic pressure, which increases tension and complicates safe stapler placement. We present a capnothorax-assisted technique and provide a video demonstration.

**Methods:**

A retrospective analysis of consecutive patients undergoing minimally invasive liver resection with en-bloc diaphragmatic resection between July 2022 and May 2025 was conducted following IDEAL recommendations. The technique consists of inducing a controlled capnothorax using CO_2_ insufflation (8 mmHg) via a Veress needle inserted into the pleural cavity under direct visualization. This neutralizes thoracic negative pressure and facilitates tension-free stapled diaphragmatic transection. A supplementary video illustrates the key technical steps.

**Results:**

Six patients underwent capnothorax-assisted diaphragmatic resection. A minimally invasive approach was completed in all cases, with one conversion unrelated to the technique. R0 resection was achieved in all patients. No intraoperative complications or pulmonary adverse events were observed. Median operative time was 473 minutes (range 288–825), and median blood loss was 10 mL (range 10–200). Median length of stay was 4 days (range 3–7), with no postoperative complications (Clavien–Dindo ≥ I). No pleural drainage was required in capnothorax cases. No diaphragmatic recurrences were observed at follow-up.

**Conclusions:**

Capnothorax-assisted diaphragmatic resection is a simple and reproducible technique that facilitates stapled en-bloc resection by transforming a traction-dependent maneuver into a pressure-neutralized approach. The accompanying video provides a practical guide for implementation. These findings are hypothesis-generating and require validation in larger comparative studies.

**Supplementary Information:**

The online version contains supplementary material available at 10.1007/s00464-026-12794-6.

Hepatic tumors located in the posterior and superior segments, particularly those abutting the hepatic dome, eventually invade the diaphragm. Achieving complete tumor resection with negative margins in these cases often requires an en-bloc resection of both the hepatic tumor and the involved diaphragm. This scenario presents significant technical challenges, especially in minimally invasive surgery, and is frequently associated with increased postoperative morbidity and prolonged hospital stay [1].

While diaphragmatic stripping—removal of only the peritoneal sheath of the diaphragm—has been utilized in cytoreductive procedures with the intent to reduce pulmonary complications [1–5]. For obvious reasons, this technique is not feasible when direct diaphragmatic invasion by the tumor is present. Under these circumstances, a full-thickness diaphragmatic resection is mandatory.

The traditional method for en-bloc diaphragm resection (in both open and minimally invasive approaches) involves direct incision and suture repair. This was our initial approach in laparoscopy (see Supplemental Video 1).

A new method for full-thickness resection was later described, involving the use of a linear stapling device, which avoids opening the pleural cavity, reduces intraoperative hemodynamic disturbance, and theoretically minimizes the risk of postoperative pneumothorax and other pulmonary complications [1–5]. However, the negative pressure within the thoracic cavity creates substantial technical difficulties [4, 5]. In order to insert the endostapler adequately, and to counteract the negative thoracic pressure, a significant traction must be applied to the blocked liver-diaphragm. This maneuver is not only technically demanding but also increases the risk of tumor rupture at the diaphragm–tumor interface and subsequent tumor spillage [2, 3, 5]. Additionally, manipulating the stapling device while maintaining adequate traction can be particularly challenging in the minimally invasive setting.

After transitioning to a stapling technique, we consistently encountered the aforementioned technical difficulties. Attempts to address these limitations through alternative approaches have been limited. While pneumothorax-induced diaphragmatic relaxation has been described in thoracic surgery for exposure purposes, its deliberate application to facilitate stapled resection in minimally invasive liver surgery has not been reported. Furthermore, the use of CO_2_ rather than air, capitalizing on its rapid absorption to avoid pleural drainage, represents a distinct technical advance.

The aim of this paper is to present a safe and reproducible technique to facilitate full-thickness, stapled phrenic resection by creating a controlled capnothorax. A method that transforms the procedure from one dependent on mechanical traction to one enabled by controlled pressure neutralization.

## Technique description and evolution

**Fig. 1:**
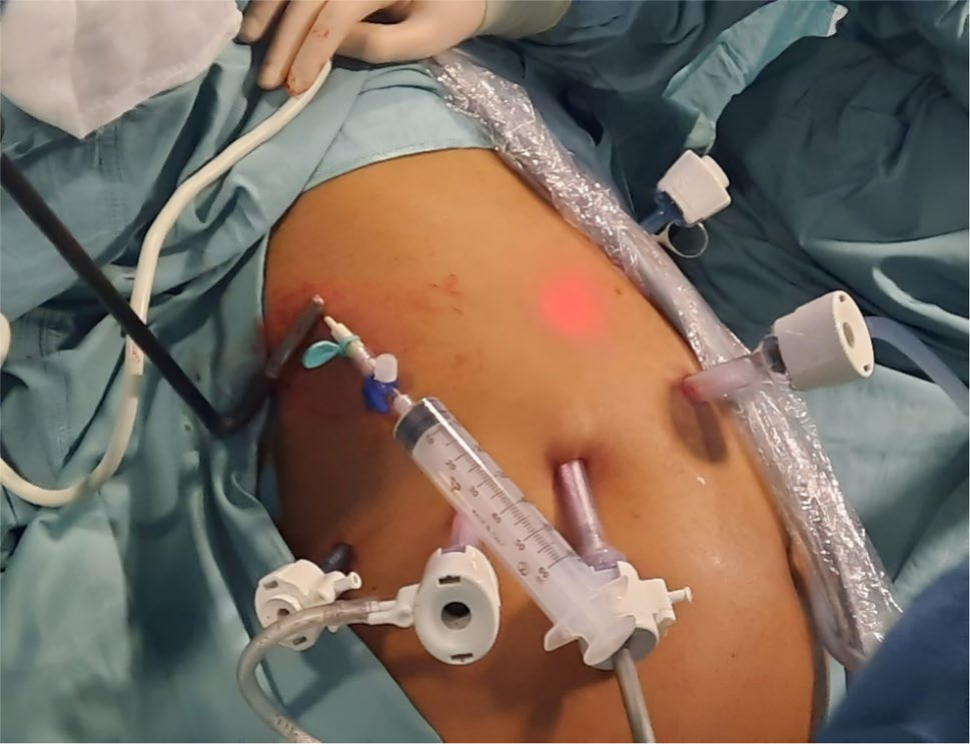
First approach: US-guided puncture

Our approach evolved through three iterations. Initial technique (July 2022, n = 2): Ultrasound-guided puncture with an 18-gauge catheter (Abocath) into the pleural space, followed by air insufflation to create pneumothorax. This facilitated diaphragmatic descent and stapler insertion. Posteriorly, air was aspirated via suction, the lung hyperinflated, and a PleurEvac drain was maintained until chest X-ray on postoperative day 1 (Fig. 1).

**Fig. 2:**
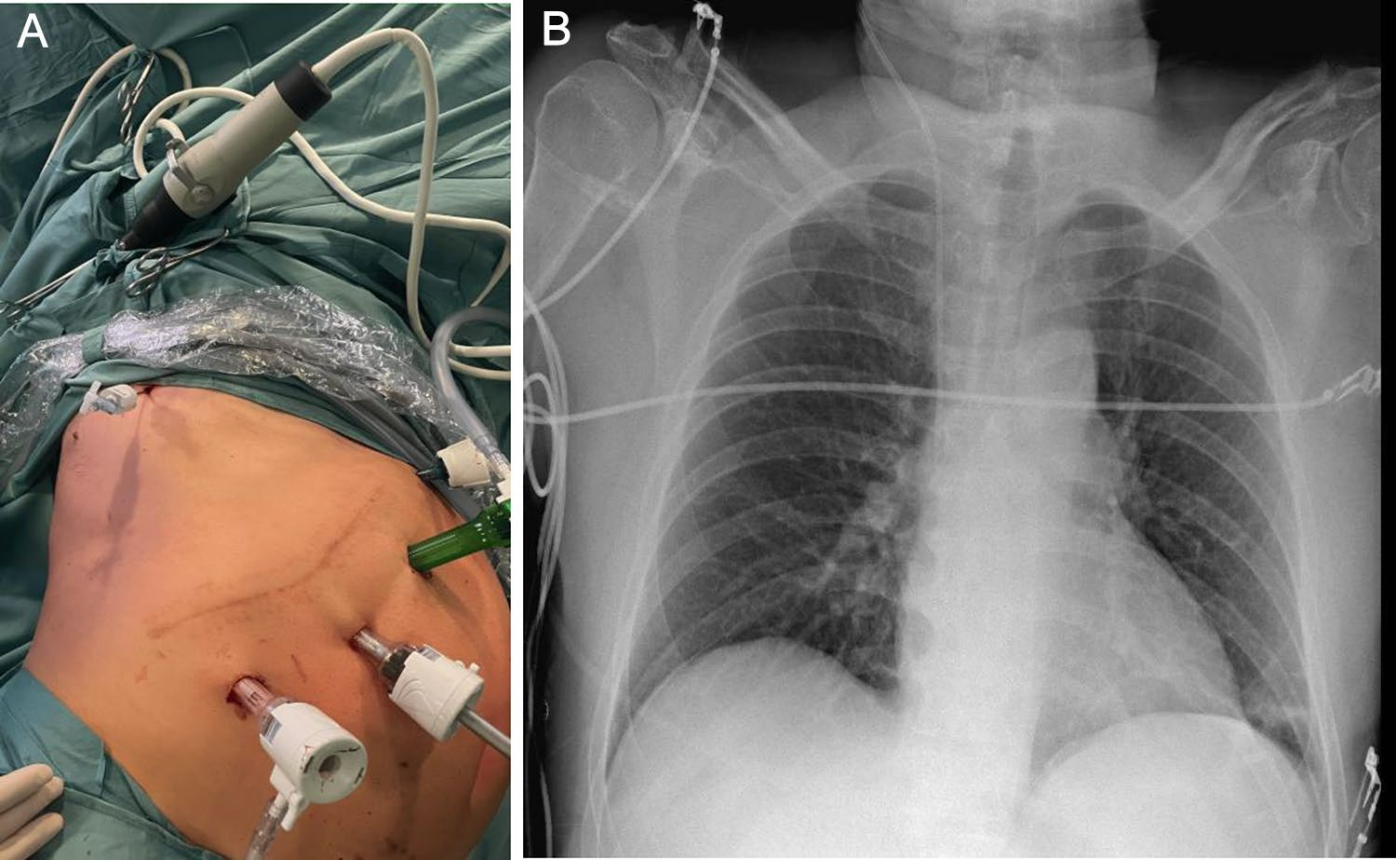
**A** Placement of the Veress needle between the 7th and 8th intercostal spaces along the midaxillary line. **B** 18-h postoperative chest X-ray revealing no evidence of residual pneumothorax

**Fig. 3:**
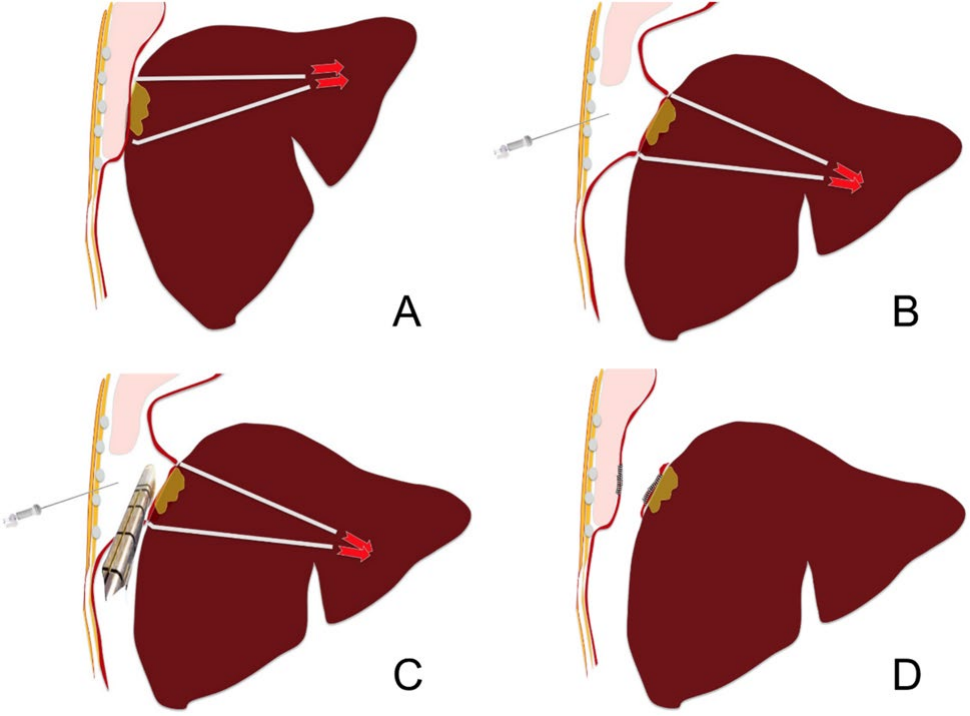
Step-by-step description of the surgical technique: **A** Tumor dissection with placement of a tape to achieve circumferential isolation and controlled traction.; **B** Creation of a capnothorax through the 7th–8th intercostal space, allowing diaphragmatic descent and improved surgical exposure.; **C** Diaphragmatic transection performed using an endoscopic linear stapler; **D** Completion of diaphragmatic resection with evacuation of the capnothorax

*Refined technique*: We subsequently introduced a Veress needle (7th–8th intercostal space, right midaxillary line) under direct visualization, initially with air (first two cases), then transitioning to capnothorax (final technique, n = 4). CO_2_ insufflation (8 mmHg) provided controlled diaphragmatic relaxation for tension-free stapler placement. Following resection, CO_2_ was evacuated during a Valsalva maneuver; the needle was removed and the incision closed immediately (Figs. 2 and 3).

*Rationale for CO*_*2*_: Its 20-fold greater solubility compared with air enables rapid absorption (30–60 min) without pleural drainage [6], eliminating the need for postoperative catheters.

### Anaesthesia perspective

With the patient fully relaxed, ventilation is maintained with 100% FiO_2_ for at least 5 min. The patient is then allowed to breathe spontaneously with an airway pressure of 0 cmH_2_O. Once the lungs are completely emptied, the surgeon punctures the thorax using the Veress needle. CO_2_ insufflation is initiated while the patient remains in spontaneous breathing, ensuring that expired CO_2_ remains at 0 mmHg. CO_2_ insufflation was pressure-controlled at 8 mmHg, selected as the lowest effective pressure for optimal stapler placement. Notably, in our series of 6 patients, this pressure was well tolerated without perturbation of pulmonary homeostasis. No patient presented contraindications requiring protocol modification, including those with mild-to-moderate pulmonary comorbidities.

Once the capnothorax is achieved, mechanical ventilation is resumed with tidal volumes of 6 mL/kg of ideal body weight, without PEEP. Expired CO_2_ is continuously monitored to detect any inadvertent pulmonary laceration, which would result in a significant increase in expired CO_2_. FiO_2_ is maintained at 100% throughout the procedure.

After the resection is completed, the patient is returned to spontaneous breathing with 0 cmH_2_O airway pressure. The surgeon then begins aspirating the pleural CO_2_, verifying that the diaphragmatic bulging into the abdomen disappears. Once no further gas is aspirated, a progressive increase in airway pressure is applied while continuing aspiration, and the needle is removed from the thorax.

### Case presentation

A 57-year-old male was diagnosed with stage-IV colon adenocarcinoma with liver involvement in August 2019. He underwent treatment with CAPOX, followed by a right hemicolectomy and simultaneous multiple atypical hepatectomies. Histological analysis of the surgical specimen revealed a low-grade enteric adenocarcinoma, staged as ypT3 ypN2b (9/21). Adjuvant treatment with Capecitabine-Oxaliplatin was administered.

In September 2021, radiological findings indicated nodal progression in the right cardiophrenic region and five new focal liver lesions located in segments II, III, and VIII. A two-stage strategy was planned to achieve complete disease clearance. The first stage focused on clearing the left hepatic lobe of disease through an anatomical resection of segment III and an atypical resection of a lesion in segment II, performed laparoscopically on February 24, 2022. This was followed by radioembolization with Y90 spheres targeting the right hepatic lobe to induce progressive hypertrophy of the left hepatic lobe and serve as a test of time.

Following radiological evidence of tumor response and adequate hypertrophy of the future liver remnant, a laparoscopic right hepatectomy was planned. During this subsequent surgery, upon completing the dissection of the right liver attached to the diaphragm’s dome and omentum, doubts arose about diaphragmatic infiltration by the tumor. To facilitate resection of the diaphragm, a controlled capnothorax was induced (See Supplemental Video 2). In this particular case, the resection was technically challenging due to the intense hilar inflammatory changes and fibrosis induced by prior radioembolization. These alterations made safe dissection of the hepatic pedicle impossible. As a result, conversion to an open approach was required. This occurred after completion of the diaphragmatic resection, which at the time of the hilar dissection had already been performed laparoscopically.

## Methods and results

This is a retrospective analysis of consecutive patients from a prospectively maintained database, conducted following IDEAL (Idea, Development, Exploration, Assessment, Long-term study) recommendations for surgical innovation [7]. Between July 2022 and May 2025, six consecutive patients underwent minimally invasive liver resection with en-bloc diaphragmatic resection using the above-described capnothorax technique at our institution. All patients with intraoperative suspicion of diaphragmatic involvement during this period were included without exclusion criteria. The demographic and clinical characteristics of these patients are presented in Table 1.

**Table 1:** Case series

Patient	Sex	Age	Type of tumor	Diaphragmatic infiltration in pathology	Technique	Placement of pleural catheter	ICU days	LOS days	Southampton Scale	IWATE scale	Blood loss (ml)	Clavien–Dindo scale	Conversion	Operative time (min)
1	Female	67	Colon adenocarcinoma	–	US-guided puncture (air)	Yes	1	5	5	5	10	0	No	441
2	Male	56	Rectal adenocarcinoma	No	US-guided puncture (air)	Yes	1	4	5	5	10	0	No	319
3	Male	42	Rectal adenocarcinoma	No	Veress needle (air)	No	1	3	7	9	10	0	No	557
4	Male	57	Colon adenocarcinoma	No	Veress needle (air)	No	3	7	7	6	200	0	Yes*	825
5	Male	64	Sclerosed hemangioma (suspected renal cell tumor metastases)	No	Veress needle (Co2)	No	1	4	9	11	50	0	No	288
6	Female	69	Hepatocarcinoma	–	Veress needle (Co2)	No	1	4	3	6	10	0	No	345

*ICU* intensive care unit, *LOS* length of stay

*Case presentation

A phrenic resection was feasible minimally invasive in all cases, and also an R0 specimen was achieved in all of them. No intraoperative complications of adverse events were observed—related or not with this technique. A chest X-ray was routinely obtained immediately after the procedure upon admission to the intensive care unit, and again on the first postoperative day, confirming satisfactory recovery in all cases (Fig. 2b). Additionally, in the first two cases in whom a pleural drain (Pleurevac) was placed, it was successfully removed on postoperative day one without adverse outcomes (Table 1).

All patients followed our ERAS protocol, which emphasized early mobilization and oral intake tolerance beginning on the day of surgery.

Perioperative outcomes and surgical complexity are presented in Table 1. Operative time was 393 min (range 288–825). Estimated blood loss was 10 mL (range 10–200). IWATE [8] difficulty score was 6 (range 5–11). Southampton [8] intraoperative adverse event grade was 6 (range 3–9). ICU stay was 1 day (range 1–3). Length of stay was 4 days (range 3–7). Postoperative complications according to the Clavien–Dindo classification [9] were Grade 0 in all six patients. Conversion to open surgery occurred in one patient (16.7%) due to reasons unrelated to the described capnothorax technique (Case presentation). At long-term follow-up, three patients developed metastatic recurrence at sites other than the liver, and three patients showed no recurrence of their primary disease.

The overall postoperative course was uneventful, with no complications, including pulmonary events. There was no need for prolonged ICU stays or increased oxygen demand.

## Discussion

The resection of the diaphragm, particularly in the context of minimally invasive liver surgery, represents a highly complex procedure, often limited to specialized centers with advanced laparoscopic expertise. This challenge is especially evident in cases of large hepatic tumors involving the superior and posterior liver segments, where achieving negative margins necessitates en-bloc diaphragmatic resection [1, 2, 4, 10–13]. The technical demands of this approach, coupled with the potential for significant postoperative complications, have historically constrained its widespread adoption [14].

However, when performed in specialized centers, en-bloc resection of the liver and diaphragm does not necessarily result in increased complications. Studies have shown that these procedures can be performed safely with acceptable morbidity and mortality rates. For instance, a study comparing patients undergoing combined liver and diaphragmatic resection with those undergoing hepatectomy alone found no significant difference in overall survival and disease-free survival between the groups, indicating that the procedure is justified when diaphragmatic infiltration is suspected [1, 2, 10–13].

Despite the feasibility of laparoscopic right hepatectomy with diaphragmatic resection, reported outcomes indicate increased operative time, higher intraoperative blood loss, and a greater likelihood of conversion to open surgery compared to procedures without diaphragmatic involvement [11, 15]. Moreover, the decision to proceed with resection is complicated by the difficulty in distinguishing true pathological diaphragmatic invasion from inflammatory adhesions intraoperatively. Studies have demonstrated that true pathological invasion is associated with a significantly higher risk of peritoneal recurrence and worse overall survival compared to cases where only inflammatory adhesions are present [11]. Our technique builds upon established stapled diaphragmatic resection methods while addressing their primary limitation. Karoui et al. [12] first described stapled resection without pleural opening; Kazaryan et al. [4] demonstrated feasibility in laparoscopy, however, neither addressed the fundamental problem of diaphragmatic tension generated by negative intrathoracic pressure. The intentional creation of capnothorax transforms this from a force-dependent to a pressure-neutralized procedure.

As in other specialized centers, we initially performed traditional en-bloc liver and diaphragmatic resections, as described in the literature [16]. This new technique represents a progressive refinement of the standard approach to diaphragmatic resection, aimed at mitigating the inherent challenges of these procedures. The controlled induction of a capnothorax using CO_2_ insufflation provides a more favorable surgical field by increasing diaphragmatic distensibility, improving tissue handling, and making an evacuation catheter unnecessary. Compared to traditional stapled or direct incision methods, our approach facilitates precise, tension-free transection, while reducing the need for complex diaphragm reconstruction and pleural drainage postoperatively. This method is particularly advantageous in cases where the diaphragmatic tissue is thick and under tension, conditions that often limit the effectiveness of conventional stapling devices.

Oncological considerations should be taken into account. An important finding of our series was that histological confirmation of diaphragmatic invasion was not obtained in any of the 6 patients (0%), despite intraoperative suspicion in all cases. While this may appear discordant, it aligns with published literature demonstrating that macroscopic adhesion frequently overestimates true pathological invasion. In contemporary series of hepatectomy with diaphragmatic resection for suspected involvement, histologically confirmed invasion occurs in only 20–30% of cases, varying by tumor type. In hepatocellular carcinoma, Liu et al. [10] reported true invasion in 21% of patients undergoing en-bloc resection, with the remainder demonstrating only fibrous adhesion without tumor infiltration. Similarly, Okuno et al. [17] described a 21% pathological invasion rate in colorectal liver metastases with suspected diaphragmatic involvement. Thus, our finding is not anomalous; rather, it reflects the inherent difficulty of preoperatively distinguishing inflammatory adhesion from true invasion in posterosuperior liver lesions, a limitation that persists despite modern imaging.

This discrepancy raises valid questions regarding the necessity of routine en-bloc resection when invasion cannot be confirmed. However, two considerations support our approach: first, intraoperative frozen section assessment of diaphragmatic adherence is technically unreliable and risks tumor violation during sampling; second, the consequences of leaving unrecognized invasion (positive margins, tumor spillage) outweigh the morbidity of resection in appropriately selected patients. Importantly, the purpose of our capnothorax technique is not to expand indications for diaphragmatic resection, but to optimize oncological execution when en-bloc resection is deemed necessary based on intraoperative assessment—ensuring negative margins and avoiding tumor violation in a technically demanding scenario. R0 resection was achieved in all our patients without tumor spillage or diaphragmatic recurrence at long-term follow-up. This distinction is critical: we offer a technical refinement for appropriately indicated cases, not a justification for more extensive resection.

The induction of capnothorax during major liver surgery warrants careful physiological monitoring, though our experience suggests it is well tolerated when appropriately managed. In our series, 8 mmHg CO_2_ insufflation preserved pulmonary homeostasis without observed hypercapnia, hemodynamic instability, or oxygenation impairment. The rapid absorption of CO_2_, 20 times more soluble than air, facilitates complete resolution without pleural drainage, contrasting with air pneumothorax techniques [18].

We did not identify specific contraindications related to pre-existing pulmonary pathology in our patient cohort; however, we acknowledge this reflects a favorable selection and a limited sample size. Contraindications for this technique are the same as those for any major minimally invasive hepatectomy. We present this as an empirically derived protocol effective under monitored conditions, requiring prospective validation for broader application.

This report has limitations*.* The small sample size (n = 6), single-center design, and absence of a control group preclude definitive conclusions regarding comparative effectiveness. The first two cases represent technical evolution (air pneumothorax with drainage), introducing heterogeneity. These findings are hypothesis-generating and require validation in larger comparative studies. However, the simplicity of the technique, with materials available in any operating room suggests its easy applicability. The advantages are evident. In our series, we have experienced how en-bloc liver–phrenic resection has turned from a challenging procedure to an easy and straightforward technique. Additionally, it provides clear benefits: we observed an absence of pulmonary complications and a streamlined postoperative recovery process, likely attributable to the avoidance of prolonged pleural exposure, the meticulous evacuation of residual CO_2_ as well as its rapid absorption following resection.

In conclusion, this report describes a refined technique for capnothorax-assisted laparoscopic diaphragmatic resection that may offer technical advantages in selected cases of suspected diaphragmatic involvement. Our preliminary experience suggests that controlled capnothorax facilitates tension-free stapled transection and may simplify the procedure in challenging anatomical scenarios. While these findings are promising, they remain hypothesis-generating and require validation in larger series with longer follow-up before any conclusions regarding safety, reproducibility, or impact on postoperative outcomes can be drawn. This approach should currently be considered within the context of specialized centers with advanced laparoscopic expertise, pending further comparative evaluation.

## Supplementary Information

Below is the link to the electronic supplementary material.

Supplementary file1 (MP4 45673 kb)

Supplementary file2 (MP4 47679 kb)
